# Development of ipilimumab: a novel immunotherapeutic approach for the treatment of advanced melanoma

**DOI:** 10.1111/nyas.12180

**Published:** 2013-06-17

**Authors:** Jedd D Wolchok, F Stephen Hodi, Jeffrey S Weber, James P Allison, Walter J Urba, Caroline Robert, Steven J O'Day, Axel Hoos, Rachel Humphrey, David M Berman, Nils Lonberg, Alan J Korman

**Affiliations:** 1 Memorial Sloan-Kettering Cancer CenterNew York, New York; 2 The Dana–Farber Cancer InstituteBoston, Massachusetts; 3 H. Lee Moffitt Cancer Center and Research InstituteTampa, Florida; 4 MD Anderson Cancer CenterHouston, Texas; 5 Earle A. Chiles Research Institute at Providence Cancer CenterPortland, Oregon; 6 Institut Gustave RoussyVillejuif, France; 7 Beverly Hills Cancer CenterBeverly Hills, California; 8 GlaxoSmithKlineCollegeville, Pennsylvania; 9 MethylGene Inc., MontrealQuebec, Canada; 10 Bristol-Myers SquibbPrinceton, New Jersey; 11 Bristol-Myers SquibbRedwood City, California

**Keywords:** cytotoxic T-lymphocyte antigen-4, immuno-oncology, immunotherapy, ipilimumab, melanoma, monoclonal antibody

## Abstract

The immunotherapeutic agent ipilimumab has helped address a significant unmet need in the treatment of advanced melanoma. Ipilimumab is a fully human monoclonal antibody that targets cytotoxic T-lymphocyte antigen-4 (CTLA-4), thereby augmenting antitumor immune responses. After decades in which a number of clinical trials were conducted, ipilimumab was the first therapy to improve overall survival in a randomized, controlled phase III trial of patients with advanced melanoma. These results led to the regulatory approval of ipilimumab at 3 mg/kg for the treatment of unresectable or metastatic melanoma. More than 17,000 patients worldwide have received ipilimumab, either as a commercial drug at 3 mg/kg or in clinical trials and expanded access programs at different doses. Consistent with its proposed mechanism of action, the most common toxicities associated with ipilimumab therapy are inflammatory in nature. These immune-related adverse events were mostly reversible when effective treatment guidelines were followed. Importantly, long-term follow-up of patients who received ipilimumab in a phase III trial showed that 24% survived at least two years, and in phase II studies, a proportion of patients survived at least five years. Evaluation of ipilimumab is ongoing in the adjuvant setting for melanoma, and for advanced disease in nonsmall cell lung, small cell lung, prostate, ovarian, and gastric cancers.

## Introduction

Melanoma is less common than other types of skin cancer, yet it is an aggressive disease that accounts for approximately 75% of deaths due to skin cancer.^1^ The incidence of melanoma has increased substantially over the last three decades, with an estimated 8,700 melanoma-related deaths in the United States in 2010^2^ and estimates of 9,480 deaths in 2013.^1^ Patients diagnosed with advanced melanoma (American Joint Committee on Cancer stage IV) have a particularly poor long-term prognosis, with approximately 75% surviving less than one year and an overall 5-year mortality rate of 90%.^3^ Until recently, median overall survival (OS) for patients with advanced melanoma was approximately eight months with traditional therapies^4^ and typically less for patients with brain metastases.^5^

Traditional treatment options for patients with advanced melanoma include surgery, radiation therapy (RT), and/or systemic therapy (i.e., chemotherapy or interleukin-2 (IL-2)–based immunotherapy).^6^ Since its approval by the U.S. Food and Drug Administration (FDA) in 1975, the chemotherapeutic agent dacarbazine (DTIC) has been the most widely used single agent for the treatment of advanced melanoma.^7^ Response rates with DTIC (or its oral analogue, temozolomide) range from 5% to 12% in recent clinical trials, but responses are generally transient.^4^ The chemotherapeutic agent fotemustine produces improved response, but not OS, rates over DTIC.^4^ IL-2, which is also approved in the United States for metastatic melanoma, can produce durable tumor responses in 5–10% of patients who may be cured of their disease.^8^ Biochemotherapy regimens with chemotherapy and traditional immunotherapies (IL-2 and interferon alpha (IFN-α)) have been extensively evaluated, but increased response rates using these regimens have not translated into improved OS.^4^–^9^ In fact, prior to 2011, no agent approved for the treatment of advanced melanoma had been shown to improve the OS in a randomized, controlled phase III trial.^4^

Progress in the treatment of advanced melanoma was based on fundamental discoveries in immunology, and specifically the identification of cytotoxic T-lymphocyte antigen-4 (CTLA-4) as a negative signaling molecule in activated T cells. This discovery led to the development of ipilimumab^10^–^11^ and tremelimumab,^12^ fully human monoclonal antibodies of the IgG1 and IgG2 isotypes, respectively, that specifically bind to CTLA-4 to augment antitumor immune responses. Ipilimumab monotherapy at 3 mg/kg (given every 3 weeks for four doses) improved the OS in a randomized, controlled phase III trial of previously treated patients with metastatic melanoma.^13^ A second randomized phase III trial with ipilimumab at 10 mg/kg plus DTIC improved the OS compared with DTIC alone in patients with treatment-naive metastatic melanoma.^14^ In a large, randomized phase III trial of tremelimumab at 15 mg/kg (once every 90 days) versus DTIC or temozolomide, there was no statistically significant difference in the OS between groups in patients with treatment-naive metastatic melanoma.^15^

**Figure 1:**
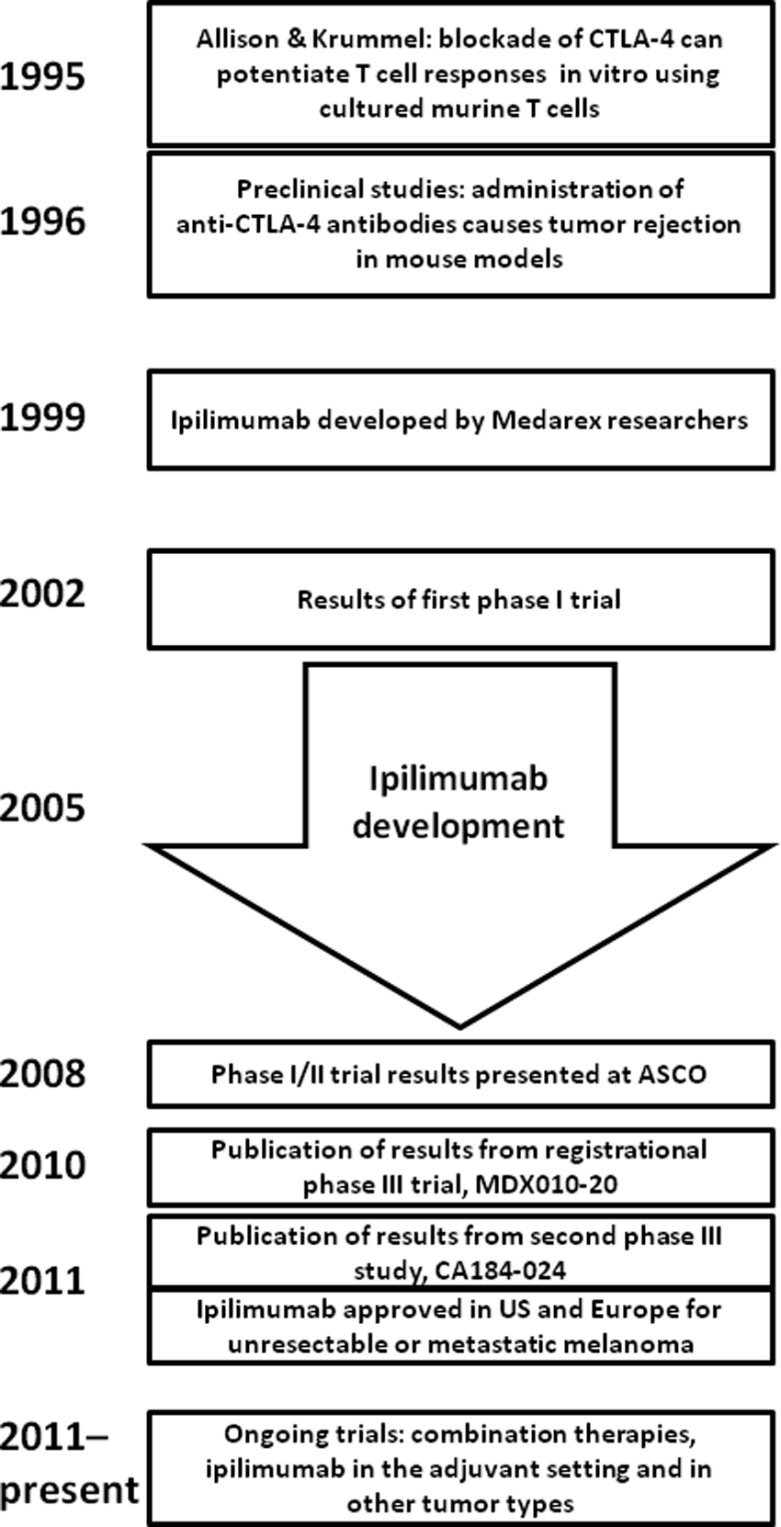
Key milestones in the development of ipilimumab. Following preclinical studies of CTLA-4 blockade in murine tumor models, ipilimumab was developed as a fully human monoclonal antibody and evaluated in several clinical trials. These trials included two randomized, controlled phase III trials in which ipilimumab demonstrated an improvement in OS. Ipilimumab received approval in 2011 in both the United States and the European Union for the treatment of patients with unresectable or metastatic melanoma. Ongoing evaluation of ipilimumab includes combination studies with other anticancer therapies (e.g., RT, anti-PD-1 antibody), as an adjuvant therapy for stage III melanoma, and its efficacy and safety in several other solid tumors.

Based on the results of the first phase III trial,^13^ ipilimumab at 3 mg/kg was approved in 2011 for the treatment of unresectable or metastatic melanoma by the U.S. FDA (treatment-naive and previously treated patients) and the European Medicines Agency (previously treated patients; Fig. 1). The scientific progress in tumor immunology, accompanied by methodological advances in trial design and clinical endpoints for immunotherapies, facilitated successful execution of the ipilimumab clinical program.^16^ Indeed, ipilimumab has made a significant impact on the treatment of advanced melanoma,^17^ and its success has ushered in a new era in the field of immuno-oncology. At the same time, advances in the understanding of aberrant molecular pathways in melanoma allowed for the development of selective inhibitors of mutated BRAF kinase.^18^ The year 2011 was one of monumental progress in the field as both ipilimumab and the BRAF inhibitor vemurafenib were approved for the treatment of advanced melanoma, ushering in an era of great hope for patients with this devastating illness.

## Immuno-oncology as a treatment paradigm

The field of immuno-oncology has evolved over more than 120 years, with several key milestones providing increasing evidence for the role of the immune system in eradicating cancer (see Refs. 19–22 for reviews on the history of immuno-oncology). One of the most significant advances occurred in 1957 when Thomas and Burnett suggested that tumor cells could evoke an immune response, and the concept of cancer immune surveillance was introduced.^22^ These and other seminal studies led to immunotherapies becoming the standard of care in some cancer types, such as Bacillus Calmette–Guerin (BCG) in superficial bladder cancer.^23^ However, early immunotherapies were limited by the lack of specificity and undefined molecular/cellular targets. Increased understanding of tumor immunology coupled with technological advances, including in methods for the discovery and production of biologics for clinical use, has allowed considerable progress in the field of cancer immunotherapy over the past three decades.^24^

Novel immunotherapeutic approaches for treating cancer were developed based on substantial evidence that the immune system is involved in tumor control^24^ and the identification of key molecules that regulate cellular immune processes.^25^ Both adaptive and innate immune systems contribute to the recognition and rejection of malignant cells,^26^,^27^ where CD4^+^ and CD8^+^ T lymphocytes play a central role in adaptive immunity.^27^ A number of molecules are known to regulate the T cell response, providing either costimulatory (e.g., CD28, 4-1BB, and OX40) or coinhibitory (e.g., CTLA-4, programmed death 1 (PD-1), and LAG-3) signals.^25^,^29^ By successfully harnessing the immune system to fight cancer, immunotherapy has become the fourth pillar (along with surgery, RT, and chemotherapy) of the cancer treatment platform.^31^

Tumor cells actively evade destruction by the immune system, which is now recognized as one of the hallmarks of cancer.^32^ Overcoming mechanisms of immune escape presents a significant challenge and may limit the effectiveness of anticancer therapies.^33^–^34^ The rationale for immuno-oncology is the potential to overcome immune escape mechanisms, to achieve long-standing antitumor effects based on the establishment of memory response, and to avoid mechanisms of resistance that occur with treatments that directly target tumors. Given their unique mechanism of action, immuno-oncology agents may have key differences from more traditional anticancer treatments with regard to the assessment of tumor response, clinical endpoints, safety management, and use in combination with other modalities.^24^–^35^

## CTLA-4 as a therapeutic target

**Figure 2:**
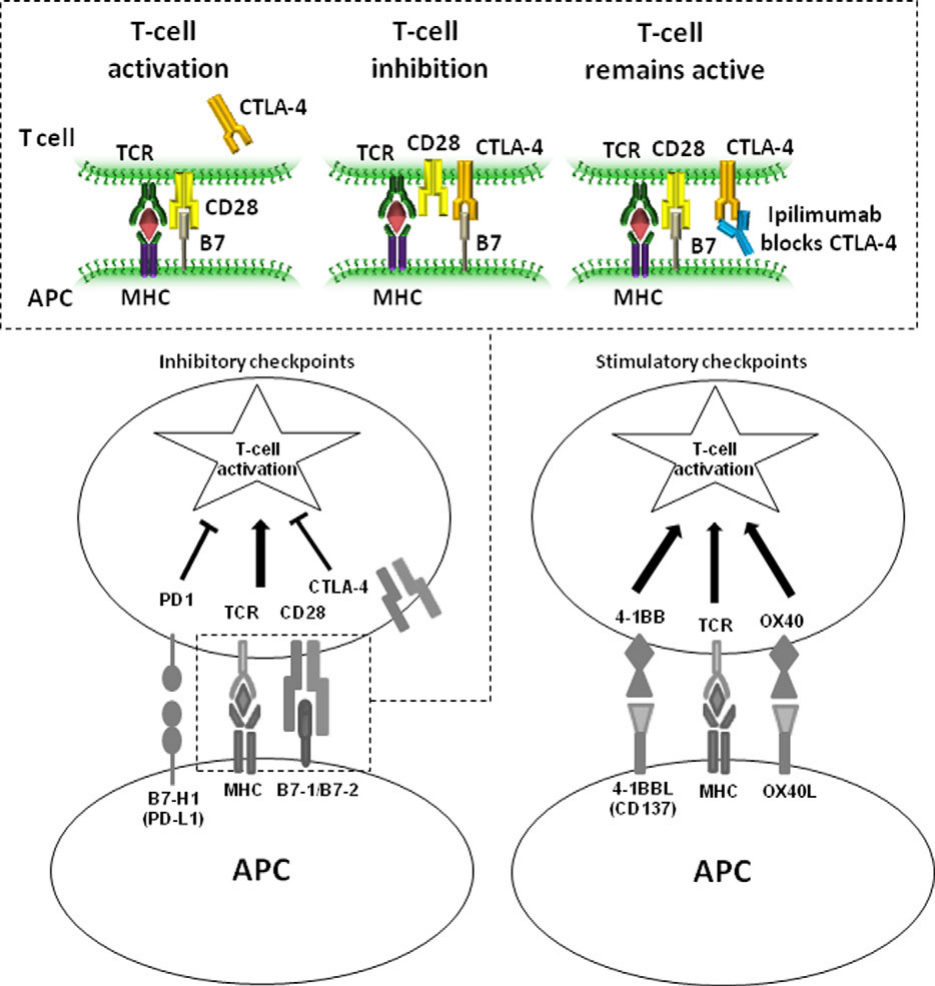
Mechanism of action of ipilimumab. Competitive inhibition of CD28-B7.1/B7.2 binding by CTLA-4 suppresses T cell activation and prevents an immune response. Blockade of CTLA-4 with ipilimumab allows continued T cell activation to augment the antitumor response. Other regulatory checkpoints with the potential for modulation include the coinhibitory molecule PD-1 as well as costimulatory molecules OX40 and 4-1BB.

Generation of an immune signal requires presentation of tumor antigen by major histocompatibility complex (MHC) class I or II molecules on an antigen presenting cell (APC). However, T cell activation and proliferation requires a second signal, typically generated when CD28 on the T cell surface simultaneously binds to a costimulatory B7-1/B7-2 ligand on the APC (Fig. 2). Following activation, T cells upregulate and translocate CTLA-4 receptor molecules to the surface, which bind B7 with a higher avidity than CD28. CTLA-4 successfully outcompetes with CD28 to generate an opposing signal that inhibits T cell proliferation and IL-2 secretion.^34^–^38^ CTLA-4 is therefore a key negative regulator of endogenous T cell–mediated responses, serving as a natural braking mechanism and allowing for a return to homeostasis following an immune response.^39^

Preclinical studies using explanted T cells in culture^40^ and mice deficient in CTLA-4^41^–^42^ established the role of CTLA-4 as a key negative regulator of T cell activation. Subsequent research that led to the discovery and development of ipilimumab was carried out by Allison *et al*.^43^ at the University of California, Berkeley, who proposed that an understanding of the role of CTLA-4 in T cell regulation might be used to design cancer therapies. They showed that a CTLA-4 blocking antibody could mediate immune rejection of even pre-established tumors in a mouse model of colon cancer.^43^ This was followed by additional studies of antibody blockade (either alone or in combination with other agents) in mouse models of melanoma and prostate cancer.^38^,^44^ Scientists at Medarex, led by Alan Korman, then developed human monoclonal antibodies that block human CTLA-4, which were generated using a transgenic mouse comprising human immunoglobulin genes.^46^ Studies in cynomolgus macaques showed that these anti-CTLA-4 antibodies could augment a vaccine response.^46^ In addition, chronic administration of one fully human anti-CTLA-4 antibody (10D1) to macaques did not result in treatment-related pathology (i.e., autoimmunity) nor did it elicit any detectable monkey antihuman antibody responses.^46^ The 10D1 (MDX-010) antibody was advanced to clinical evaluation and later assigned the United States Adopted Name (USAN) or generic name of ipilimumab.^10^

## Clinical evaluation of ipilimumab in phase I/II trials

Early clinical evaluation of ipilimumab included several tumor types, such as prostate cancer and advanced melanoma. The initial studies in patients with melanoma evaluated ipilimumab at single or multiple doses ranging from 0.1 to 20 mg/kg, either as monotherapy or in combination with IL-2,^47^ gp100 melanoma peptide vaccine,^48^ or chemotherapy.^49^ These studies documented objective responses with ipilimumab in patients with melanoma, which were durable in many cases. In a phase I/II study of metastatic castration-resistant prostate cancer (mCRPC), in which 50 patients received ipilimumab at 10 mg/kg ± RT, eight had prostate-specific antigen (PSA) declines of at least 50%, one had a complete response, and six had stable disease.^50^ The initial characterization of ipilimumab's safety profile identified the inflammatory nature of treatment-related adverse events (AEs), which were presumed to reflect ipilimumab's immune-based mechanism of action.

A series of phase II studies evaluated ipilimumab in more than 500 patients with advanced melanoma.^51^–^54^ Efficacy and safety were evaluated in treatment-naive and previously treated patients in study CA184-007, and in previously treated patients in study CA184-008. Study CA184-022 was a dose-ranging trial in patients that had been previously treated or were intolerant to prior therapy. The OS was a secondary endpoint in each of these studies. Another trial (CA184-004) was an exploratory biomarker study with ipilimumab in treatment-naive and previously treated patients. In all studies, ipilimumab was given intravenously every 3 weeks for four doses (induction), and eligible patients could receive maintenance therapy every 12 weeks beginning at week 24.

Two key lessons were derived from the outcomes of these phase II studies. First, they identified immune-related AEs (irAEs) as the most common toxicities associated with ipilimumab treatment, which were confirmed by histopathology as inflammatory in nature. The irAEs were shown to be reversible in most cases following adherence to treatment guidelines developed during the phase II studies (see below). Second, these studies documented the unique kinetics of response to ipilimumab, where some patients showed responses after apparent disease progression, possibly reflecting the time required to establish antitumor immunity. Objective responses were sometimes observed 6–12 months after the initiation of treatment. Prolonged stable disease (≥1 year) likely reflected an additional characteristic of ipilimumab efficacy. Furthermore, it was shown that these response kinetics may translate into a delayed separation of survival curves, a previously described feature of immunotherapy needing special consideration for phase III studies.^16^–^35^ Thus, it was recognized that the OS was a superior endpoint to capture the clinical activity of ipilimumab, and the primary endpoint for both phase III trials was changed to OS (prior to unblinding). Finally, biomarker analyses confirmed that ipilimumab treatment increases activated and memory T cell populations,^55^ consistent with its proposed mechanism of action.^10^

## Phase III trials of ipilimumab in advanced melanoma

**Figure 3:**
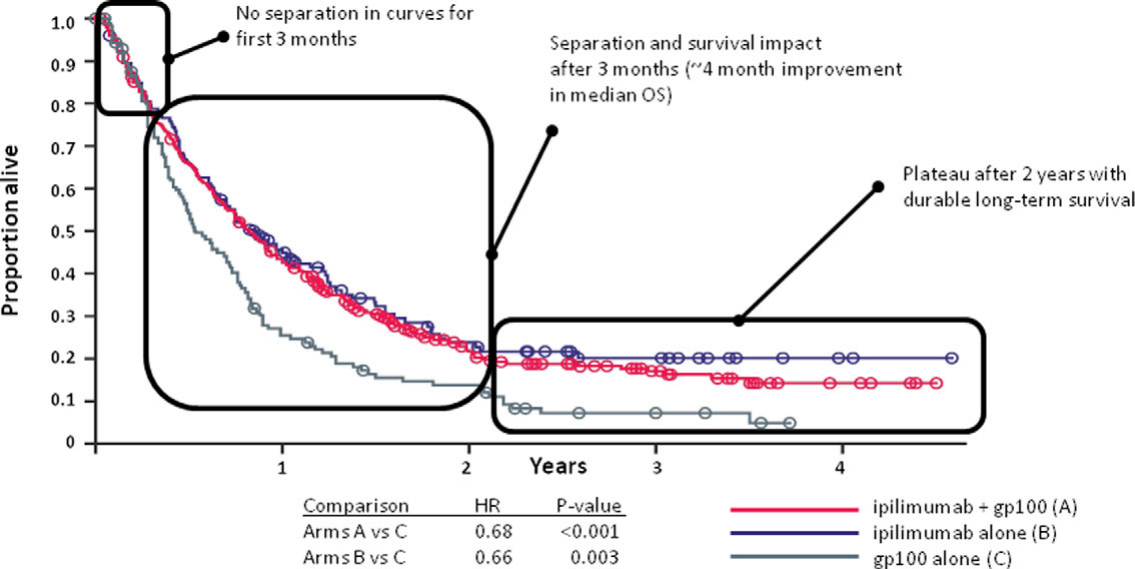
Kaplan–Meier analysis of overall survival in the phase III study MDX010-20. Separation of the Kaplan–Meier survival curves was not observed until three months, at which time an OS benefit of approximately four months was observed with ipilimumab treatment compared with the gp100 control. Survival in the ipilimumab monotherapy group reached a plateau after two years, indicating durable response and long-term survival benefits. The table shows data for the primary endpoint of OS, and secondary endpoints of 1- and 2-year survival rates. Modified, with permission, from Ref. 13.

The phase III trial, MDX010-20,^13^ began in 2003 with ipilimumab treatment at 3 mg/kg based on evidence of safety and activity in early clinical trials.^10^ Previously treated patients who received ipilimumab, with or without gp100 as an active control, had significantly improved OS compared with those who received gp100 alone (Fig. 3).^13^ A near doubling of 1- and 2-year survival rates with ipilimumab compared to gp100 was observed (Fig. 3). Importantly, clinical outcome was independent of the stage at presentation (M0, M1a, and M1b vs. M1c) and baseline lactate dehydrogenase (LDH) levels, both of which are important prognostic factors for melanoma, as well as age (<65 vs. ≥65 years). Prior treatment with IL-2 also did not preclude a response to ipilimumab. The frequency of irAEs was approximately 60% in patients who received ipilimumab, with 10–15% having melanoma of grade 3 or 4. The results of study MDX010-20 were the basis for regulatory approval of ipilimumab at 3 mg/kg for previously treated metastatic melanoma and, in some countries (including the United States), for treatment-naive metastatic melanoma.

**Figure 4:**
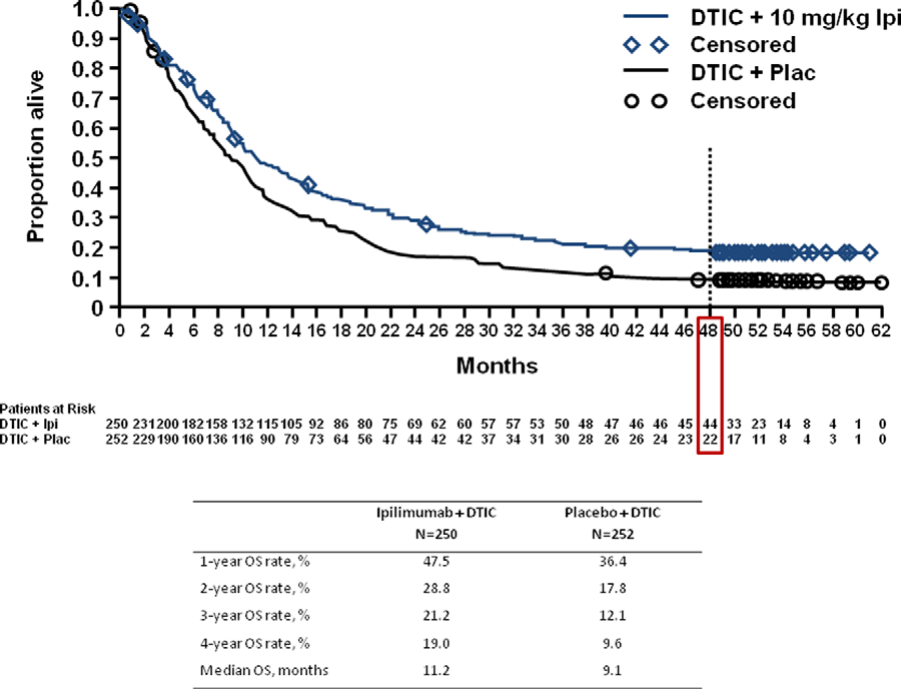
Kaplan–Meier analysis of overall survival in the phase III study CA184-024. Survival analysis of OS in treatment-naive patients with advanced melanoma who received ipilimumab at 10 mg/kg plus DTIC or placebo plus DTIC in the phase III trial, CA184-024. The survival curves reach a plateau beginning at approximately three years after initiation of treatment. Continued survival follow-up of more than four years demonstrates a long-term survival benefit that is consistent with the results of other ipilimumab studies. The table shows data for the primary endpoint of OS, with secondary endpoints of 1-, 2-, and 3-year survival rates as originally reported^14^ and 4-year survival rates based on recent survival follow-up.^64^

The results of another phase III study, CA184-024, showed that ipilimumab can also significantly improve survival when used in treatment-naive patients with advanced melanoma. In this study, patients were randomly assigned 1:1 to receive either ipilimumab (10 mg/kg) plus DTIC (850 mg/m^2^) or DTIC plus placebo (control arm).^14^ The OS was significantly longer in the ipilimumab plus DTIC group than in the DTIC plus placebo group (Fig. 4) and there was a 24% reduction in the risk of disease progression with the addition of ipilimumab to DTIC. The types of AEs were consistent with those observed in studies of ipilimumab monotherapy; however, the incidence of hepatic AEs was more frequent than expected for ipilimumab alone at 10 mg/kg.^14^ This was potentially due to the combination with DTIC, which alone is associated with low-level hepatotoxicity.^56^–^57^

## Activity of ipilimumab in key patient subsets

In addition to the patient subsets described previously for the phase III trials, ipilimumab has demonstrated activity in patients with melanoma brain metastases and in melanoma patients with mutations in the BRAF kinase. BRAF-activating mutations, which drive uncontrolled cell proliferation, are known to occur in 50–60% of melanomas.^58^ In the phase II study CA184-004, similar rates of objective response and disease control were reported with ipilimumab in patients with and without the BRAF V600E mutation,^59^ suggesting that ipilimumab has activity in advanced melanoma independent of BRAF mutation status.

An open-label, phase II trial (CA184-042) was conducted to evaluate ipilimumab at 10 mg/kg in 72 melanoma patients with asymptomatic (stable) or symptomatic (active) brain metastases.^60^ Importantly, rates of objective response or stable disease were similar in the brain and in visceral lesions for both cohorts, suggesting that the blood–brain barrier does not restrict ipilimumab's activity (via T cells) as it does with some anticancer agents. There was a low incidence of central nervous system (CNS) AEs, the most common being grade-1 or -2 headache and dizziness, possibly related to ipilimumab. Recently reported results from the phase II NIBIT-M1 trial, which evaluated ipilimumab plus fotemustine in 20 patients with melanoma brain metastases, showed a median OS of 13.4 months and a 1-year survival rate of 54.2%.^61^ Collectively, these results describe the safety and activity of ipilimumab in melanoma patients with brain metastases.

## Long-term survival benefit with ipilimumab

The portfolio of phase II trials provide the longest follow-up times for ipilimumab, which have exceeded 5 years in several studies (Table1). Five-year survival rates for previously treated patients who received ipilimumab at 10 mg/kg ranged from 18.2% in study CA184-008 to 28.4% in study CA184-007, and was 16.5% for those who received ipilimumab at 3 mg/kg in study CA184-022 (with some patients being re-treated at 10 mg/kg in a rollover study).^62^ Treatment-naive patients who received ipilimumab at 10 mg/kg in study CA184-007 had 5-year survival rates of up to 49.5%. Updated survival data were also recently reported for patients treated at the U.S. National Cancer Institute in trials MDX010-05, MDX010-13, and MDX010-19.^63^ The 5-year survival rates in these studies, where ipilimumab was evaluated alone or in combination with IL-2 or gp100 peptides, were 13%, 25%, and 23%, respectively. Notably, long-term survivors included those without an objective response to ipilimumab.^62^–^63^

**Table 1:** Long-term survival of patients who received ipilimumab in phase I/II trials

	Survival rates, %				
	Median OS, months	BORR, %	3 years	4 years	5 years
*CA184-007 (n = 115)*					
10 mg/kg lpilimumab + placebo (*n* = 57); PT or TN	19.3	15.8	34	32	32
10 mg/kg ipilimumab + budesonide (*n* = 58); PT or TN	17.7	12.1	39	36	36
*CA184-008 (n = 155)*					
10 mg/kg ipilimumab; PT	10.2	5.8	23	20	18
*CA184-022 (n = 217)*					
0.3 mg/kg ipilimumab (*n* = 73); PT	8.6	0	14	14	12
3 mg/kg ipilimumab (n = 72); PT	8.7	4.2	20	18	17
10 mg/kg ipilimumab (n = 72); PT	11.4	11.1	25	22	22
*MDX010-05 (n = 56)*					
Ipilimumab + gpl00; PT (76%)	14	13	–	–	13
*MDX010-13 (n = 36)*					
Ipilimumab + IL-2; PT (66%)	16	25	–	–	25
*MDX010-19 (n = 85)*					
Ipilimumab (DE) ± gpl00; PT (94%)	13	20	–	–	23

DE, dose escalation; PT, previously treated; TN, treatment-naïve.

The survival curves show a plateau beginning at approximately three years after the start of treatment, which was observed in several phase II and III studies of ipilimumab.^62^,^63^ These findings are encouraging given that only about 10% of patients with advanced melanoma have historically survived to 5 years.^4^ Overall, these results demonstrate the most important clinical benefit with ipilimumab, namely, that a proportion of advanced melanoma patients who receive this therapy survive long term.

## Adverse events associated with ipilimumab

Across clinical studies, irAEs were the most common treatment-related AEs with ipilimumab.^65^ The majority emerge during the first 12 weeks of therapy (induction dosing period) and may involve the gastrointestinal (GI) tract, skin, endocrine, liver, or other organ systems (Table2).^65^ With ipilimumab monotherapy, the most common irAEs affect the GI tract (diarrhea/colitis) and skin (rash, pruritus). Close collaboration between Bristol-Myers Squibb and the clinical trial investigators led to the development of effective treatment guidelines for the management of irAEs.^65^,^66^ These guidelines emphasize early diagnosis and appropriate treatment as essential to minimizing severe complications. Symptomatic treatment is recommended for mild irAEs, while management of more severe reactions may require delay or omission of a dose and intense monitoring. In the case of persistent or high-grade irAEs, ipilimumab should be permanently discontinued and patients should be treated with high-dose systemic corticosteroids (with slow taper to reduce the risk of symptom relapse).^65^,^66^ Patients refractory to steroid treatment may require alternative immunosuppressive therapies.^65^ Clinical studies have shown that most irAEs are reversible when these treatment guidelines are followed (Risk Evaluation and Mitigation Strategy in the United States, Risk Management Plan in the European Union and Australia). Times to resolution for the most common irAEs typically range from 4 to 9 weeks depending on the organ system involved. Although endocrinopathies with ipilimumab treatment are uncommon, they include serious AEs such as hypophysitis (which occur in approximately 1–2% of patients) that may require long-term hormone replacement therapy.^65^

**Table 2:** Most common irAEs^a^, by organ system, with ipilimumab monotherapy at 3 mg/kg in phase III study MDX010-20 and in phase II studies 004 and 022 (pooled)

	IrAEs (%) in phase III study (*N* = 131)		IrAEs (%) in phase II studies (*N* = 111)	
Organ system affected	All grade	Grade 3-4	All grade	Grade 3-4
Any irAE	59.5	13.0	61.3	6.3
Skin	42.0	0.8	42.3	0.9
Gastrointestinal	28.2	7.6	30.6	4.5
Endocrine	7.6	3.8	4.5	0.9
Liver	3.1	0	0	0
Other	3.8	1.5	1.8	0

a AEs of an inflammatory nature that are considered causally related to ipilimumab.

## Response patterns and kinetics of activity

Ipilimumab's unique mechanism of action has implications for tumor assessments. Because of the time required to establish an antitumor immune response, it can take longer for a detectable response to evolve with ipilimumab compared to cytotoxic agents, during which time the disease may progress or appear to progress.^68^,^69^ Four distinct patterns of response have been observed with ipilimumab: (1) regression of baseline lesions with no new lesions; (2) stable disease followed by a slow, steady decline in tumor burden; (3) delayed response after an initial increase in tumor burden; and (4) response after the appearance of new lesions. The latter three response patterns are not observed with cytotoxic agents, and although they occur in <10% of ipilimumab-treated patients, they may be associated with favorable survival outcomes.^70^

The different responses observed with ipilimumab treatment should be considered in both the timing and interpretation of tumor assessments. All four induction doses should be given as tolerated, and tumor assessments should not be conducted until the end of the induction dosing period (week 12), unless there is clear evidence of clinical deterioration or disease progression. In clinical studies, it was recommended that responses be confirmed with a second assessment conducted at least 4 weeks later.^70^ The importance of this particular guideline was highlighted in the phase III MDX010-20 study, in which some patients had improved responses over time without further treatment (delayed responses).^13^

Once it was appreciated that the clinical response to ipilimumab could take time to develop, establishing guidelines for patient care and providing tools to assess efficacy more accurately became a priority. To this end, novel, exploratory immune-related response criteria (irRC), developed from modified World Health Organization (mWHO) criteria,^71^ were proposed that allow for transient increases in tumor volume or new lesions, in contrast to standard response criteria that define disease progression as the presence of new or progressing lesions.^71^–^72^ The irRC have been used in ipilimumab clinical trials of advanced melanoma and lung cancer,^61^,^70^ and have not yet been prospectively validated. While evaluation is ongoing, their use has emphasized the different response patterns with ipilimumab and the importance of confirming disease progression prior to switching therapy in asymptomatic patients.

## Further development of ipilimumab in melanoma

Ipilimumab monotherapy continues to be evaluated as a treatment for advanced melanoma and also as a potential therapy for melanoma in the adjuvant setting. Based on the results of the phase II study CA184-022, where ipilimumab at 10 mg/kg produced greater tumor (radiographic) response rates (albeit with higher frequencies of irAEs) than the approved dose of 3 mg/kg,^52^ clinical trials to further evaluate ipilimumab in melanoma and other tumor types utilize the investigational dose of 10 mg/kg. A phase III trial (CA184-169) was initiated in 2012 to determine if ipilimumab at 10 mg/kg provides superior OS than 3 mg/kg in patients with advanced melanoma.^74^

As discussed previously, ipilimumab appears to be activity independent of BRAF mutation status, and neither of the phase III trials in which ipilimumab improved OS screened patients for mutant BRAF. The BRAF inhibitor vemurafenib was approved in the United States in 2011 for patients with previously untreated, unresectable, or metastatic melanoma harboring the BRAF V600E mutation based on an improvement in OS versus DTIC in a phase III trial.^18^–^58^ With the regulatory approvals of ipilimumab and vemurafenib, critical questions have arisen for melanoma specialists regarding the use of these agents in order to optimize the benefit-risk profile for patients that harbor a BRAF mutation. Thus, ongoing or planned studies will investigate the optimal approach to using ipilimumab and BRAF inhibitors in melanoma patients with mutant BRAF.^75^–^76^

Two ongoing phase III trials are evaluating ipilimumab monotherapy as an adjuvant treatment for melanoma. Study CA184-029, conducted by the European Organization for Research and Treatment of Cancer (EORTC), will determine if ipilimumab improves recurrence-free survival versus placebo in patients with high-risk stage III melanoma rendered disease free by surgery.^77^ This study includes a standard induction phase for ipilimumab, but eligible patients can receive extended maintenance therapy with ipilimumab at 10 mg/kg, which is given every 12 weeks beginning at week 24 until disease progression or until year three. A study being conducted by the U.S. Eastern Cooperative Oncology Group (E1609) will compare recurrence-free survival and OS between ipilimumab (at 3 or 10 mg/kg) and high-dose IFN-α2b in patients with high-risk stage III or IV melanoma removed by surgery.^78^

Clinical trial data support the use of ipilimumab in combination with cytotoxic agents, particularly chemotherapy. Emerging evidence suggests that RT can activate the immune system,^79^ and thus may also be an effective partner for ipilimumab in cancer treatment. However, the timing of cytotoxic agents relative to ipilimumab may be a critical factor. For example, one study in a transgenic mouse model of prostate cancer showed that administering a tumor vaccine 3–5 weeks after RT caused an antitumor T cell response, but not when given earlier.^80^ This result suggests that RT primes the immune system to allow for greater augmentation of the antitumor immune response by immunotherapy. Interestingly, recent evidence suggests that the abscopal effect, in which local RT causes tumor regression at a distant site, contributes to the efficacy of ipilimumab and RT in advanced melanoma and that this could be mediated by an immune response.^81^ Various studies are planned to learn more about the combination of ipilimumab and RT, including an ongoing phase III trial in prostate cancer (see below).

In addition, several studies are evaluating the use of ipilimumab combined with other immunomodulatory agents. For example, possible synergistic effects of ipilimumab and intratumoral IL-2 are being evaluated in an ongoing phase II trial in advanced melanoma.^82^ The possibility of modulating multiple immune regulatory checkpoints also holds much potential. Recently, the results of a phase Ib trial were reported showing the efficacy and safety of an antibody that specifically blocks PD-1 in patients with nonsmall cell lung cancer (NSCLC), melanoma, and renal cancer.^83^ An ongoing dose-escalation phase I trial is evaluating the combination of the anti-PD-1 antibody and ipilimumab in patients with advanced melanoma.^84^

## Evaluation of ipilimumab in other tumor types

Early clinical studies suggested that ipilimumab is active against other tumor types, most notably NSCLC, SCLC, and prostate cancer.^10^ In contrast to their efficacy in melanoma, renal cell carcinoma, or metastatic prostate cancer,^11^ immunotherapies have had little or no success in NSCLC or SCLC.^85^ The rationale for using immunotherapy in lung cancer is partly based on the observation that increased tumor infiltration of T lymphocytes is associated with improved prognosis in patients with NSCLC.^85^ In the phase II trial CA184-041, ipilimumab at 10 mg/kg in a phased schedule with paclitaxel and carboplatin (PC), but not when given concurrently, significantly improved progression-free survival (PFS; by irRC or mWHO criteria) compared to PC alone in patients with untreated NSCLC^73^ and PFS (by irRC) in patients with untreated extensive-disease SCLC.^86^ Ongoing phase III trials for ipilimumab include a study comparing ipilimumab plus etoposide and platinum therapy to etoposide and platinum therapy alone in newly diagnosed patients with extensive-disease SCLC,^87^ and a study in untreated patients with squamous NSCLC comparing OS between ipilimumab plus PC and placebo plus PC.^88^ Based on the results of study CA184-041, a phased treatment schedule (chemotherapy initiated prior to ipilimumab) is being utilized in the phase III trials.

The early clinical studies providing evidence for the efficacy and safety of ipilimumab in patients with mCRPC led to the initiation of two phase III trials in these patients. In the phase I/II trial CA184-017, ipilimumab at doses up to 10 mg/kg (with or without a single dose of focal RT) resulted in PSA declines of ≥50% in 22% of patients with mCRPC.^50^ In ongoing phase III trials, ipilimumab (10 mg/kg) versus placebo is being evaluated in chemotherapy-naive patients with asymptomatic or minimally symptomatic mCRPC (CA184-095), and following a single dose of bone-directed RT in patients with mCRPC that have received prior docetaxel (CA184-043).^10^ Investigations of ipilimumab have also been undertaken in other solid tumors, including recurrent platinum-sensitive ovarian cancer,^89^ unresectable or metastatic gastric cancer following chemotherapy,^90^ and in combination with chemotherapy for metastatic bladder cancer.^91^

## Conclusions and future directions

Ipilimumab has helped address a significant unmet need for the treatment of advanced melanoma, and helped to further establish the importance of immunotherapy as an anticancer treatment. The successful development of a novel immunotherapeutic agent such as ipilimumab required close collaboration between the sponsoring pharmaceutical companies and the clinical trial investigators to better understand its unique mechanism of action, identify patterns of response, and to develop guidelines to manage its unique toxicities. Important research continues with ipilimumab, including evaluation of ipilimumab in combination with other therapies (such as anti-PD-1) to potentially increase the numbers of patients that experience clinical benefit. Ongoing phase III trials will determine if the activity of ipilimumab in advanced melanoma extends to earlier stages of disease and to other tumor types. Ipilimumab has supported the development of the immuno-oncology discipline, and CTLA-4 will hopefully be the first of many targets to be successfully exploited to fight cancer in the future.
